# Magnitude and factors associated with intimate partner violence in mainland Tanzania

**DOI:** 10.1186/s12889-016-3161-3

**Published:** 2016-06-10

**Authors:** Method R. Kazaura, Mangi J. Ezekiel, Dereck Chitama

**Affiliations:** Department of Epidemiology/Biostatistics, Muhimbili University of Health and Allied Sciences, P. O. Box 65015, Dar es Salaam, Tanzania; Department of Behavioural Sciences, Muhimbili University of Health and Allied Sciences, Dar es Salaam, Tanzania; Department of Development Studies, Muhimbili University of Health and Allied Sciences, Dar es Salaam, Tanzania

**Keywords:** Intimate partner, Tanzania, Violence

## Abstract

**Background:**

In Tanzania like in many sub-Saharan countries the data about Intimate Partner Violence (IPV) are scarce and diverse. This study aims to determine the magnitude of IPV and associated factors among ever partnered women in urban mainland Tanzania.

**Methods:**

Data for this report were extracted from a big quasi-experimental survey that was used to evaluate MAP (MAP - Men as Partners) project. Data were collected using standard questions as those in big surveys like Demographic and Health Surveys. Data analyses involved descriptive statistics to characterize IPV. Associations between IPV and selected variables were based on Chi-square test and we used binary logistic regression to assess factors associated with women’s perpetration to physical IPV and Odds Ratio (OR) as outcome measures with their 95 % confidence intervals (CI).

**Results:**

The lifetime exposure to IPV was 65 % among ever-married or ever–partnered women with 34, 18 and 21 % reporting current emotional, physical and sexual violence respectively. Seven percent of women reported having ever physically abused partners. The prevalence of women perpetration to physical IPV was above 10 % regardless to their exposure to emotional, physical or sexual IPV.

**Conclusions:**

IPV towards women in this study was high. Although rates are low, there is some evidence to suggest that women may also perpetrate IPV against their partners. Based on hypothesis of IPV and HIV co-existence, there should be strategies to address the problem of IPV especially among women.

## Background

There is multiplicity of definitions for intimate partner violence. Nevertheless, the common understanding of IPV includes all physical, sexual, or psychological harms aggravated by a current or former partner. IPV includes also threats of acts and coercion or arbitrary deprivations of liberty that may occur in public or someone’s private life perpetrated by the partner [1]. Although studies on IPV suggest its global prevalence to range between 15 % to more than 70 % among women of reproductive age (15 to 49 years), it is widely considered to be about around 30 % [2, 3]. A very recent study with large data for several sub-Saharan Africa countries reported about 40 % of women to have been exposed to some form of IPV during their life-time [4].

In Tanzania, the reported life-time prevalence of IPV ranges between 15 and 60 % [5–8]. A multi-country study conducted in 2005 gives the prevalence of lifetime physical and sexual violence by an intimate partner among ever-partnered women of 33 and 23 % respectively [9]. Furthermore, in the recent (2010) national estimates using Demographic and Health Survey (DHS), 39 and 20 % of women aged 15–49 were reported having experienced physical and sexual IPV respectively since age 15 [10].

Factors associated with violence, specifically against women, are diverse and always inter-woven. For that matter, an integrated ecological framework to understand violence against women has been developed [11]. With this model, several levels have been suggested that include those at individual, family relationships, community and societal. For example, at the individual level, the risk factors include history of violence in the perpetrator or victim’s family, male alcohol use and young age. At the family relationship level, examples of risk factors for IPV include marital conflict and male dominance in the family. Examples of community level risk factors include poverty and weak community sanctions against violence. At the societal level, examples of risk factors for violence include traditional gender norms that give men economic and decision making power in the household, and social norms that justify violence against women. These risk factors at different levels interact with each other to explain IPV. Therefore, it may be incorrect to single out one or few factors to influence IPV in a given society. Nevertheless, some scholars have argued that in many societies especially of sub-Saharan countries and other less developed nations, structural inequalities between women and men produce economic dependency among women; low women’s education status, poor social support and high parity that subject women to be at elevated risk of IPV [12, 13]. While in a multi-country study in eight African countries, Andersson et al. suggest multiple partner relationships to be associated with IPV, the study found no association with age, education level and the size of household [14]. Contrary to Andersson el at study, some studies have found increased risk of IPV with education and age [15–17]. Furthermore, alcohol consumption has been cited a risk factor for IPV [18, 19]. Among reported benefits of preventing and reducing IPV among women include a reduction of morbidities and disabilities among victims, their families, communities and the society [20]. In Tanzania, there is a limited national representative sample on IPV except a large sample [10] that reports on gender based violence. Therefore, with this study we aimed to determine the magnitude of and factors associated with IPV among women in urban mainland Tanzania using a more geographically diverse sample.

## Methods

### Study settings

The United Republic of Tanzania is a union between the mainland and the Archipelago of Zanzibar and Pemba. Tanzania mainland has a total population of 43.6 million of which 33.9 and 33.0 % of the rural population aged between 25 and 50 years are males and females respectively [10]. By the time of the study, Tanzania Mainland had 24 Regions forming 70 Districts. We selected six regions (Dar es Salaam, Kagera, Mbeya, Mwanza, Tabora and Ruvuma) to represent Mainland Tanzania. From each region, we purposefully selected one urban district (later to be used by CHAMPION – *Channeling Men’s Positive Involvement in a National HIV/AIDS Response* to train men and women on reducing HIV risk, improving reproductive health outcomes and to increase gender-equitable norms and behaviors. We further selected two wards (a ward is close to the lowest Tanzania government administrative structure at the community level that represent between 1,000 and 21,000 people. In urban settings, wards represent a portion of a town or of a larger city).

### Study design

Data for this report are extracted from a cross-sectional study to evaluate CHAMPION’S (MAP - Men as Partners) project in which the study design was quasi experimental – two arms (experimental and control). This design was applied because of its simplicity to implement. Due to logistical challenges, the two groups (an experimental and a comparison) were not assigned randomly. However, in both groups, we conducted the pre-test to both groups before conducting the training to the experimental group only. Then, a post-test was performed to assess if there were any behavior changes between and within the study groups.

We estimated a sample of 1,620 independent (not pairs) adults (aged between 25 to 50 years) women and men. Eligibility criteria included having been ever-partnered. This sample size was calculated for a quasi experimental design in order to detect at least a 10 % difference between study arms with 80 % power, a 95 % confidence interval, and accounting for a 10 % loss to follow-up. Although the main focus was men, a ratio of one woman to 2 men per site (a street and eventually a ward - an administrative unit below the district) was used in order to gather information related to women and men. Therefore, in each ward we selected streets randomly and then we systematically (based on the number of households) selected a household targeting eligible women and men in a zigzag pattern (left and right of the street) to get two men and one woman until the required sample size is attained. For the purpose of this paper, we use a sub-sample of women only.

### Process

Each respondent was invited to participate after signing the consent form. We ensured safety of both study participants and of the interviewer. Also, participants were assured of a respectful and non-threatening participation and freedom to withdraw from participating in the interview participation was voluntary and a participant can withdraw at any time. No personal identifiers were collected from any study participant. Also to enhance freedom of expression, all interviews were sex-matched; a female participant was interviewed by a female interviewer. All interviews were conducted in a strict private room close to or in the house of a study participant with a calm environment to allow both freedom of expression and to enhance confidentiality.

### Measures

Variables that were collected included background information (age, sex, marital and education status, and occupation) of the respondent and reported incidences of intimate partner violence (physical, sexual and emotional). The components for the assessment of IPV were threats and actual physical violence, sexual and emotional violence by a partner: currently (within past one month) and beyond one month but within past three months. The lifetime violence was measured by asking whether the woman ever experienced violence from the current or any previous partner. Furthermore, since also men are potentially victims of IPV, we asked if the woman ever physically hit or slapped or did anything that could harm her partner even when the partner was not already abusing them.

### The tool and assessment of IPV

The questionnaire with structured questions was used to capture information on IPV among partners. Key questions on IPV were adopted from the standard Demographic and Health Survey (DHS) tool with minor modifications to suit the setup. For example:

*Does or did the partner ever:*

- *Say or do something to humiliate you in front of others?*
- *Threaten to hurt or harm you or someone close to you?*
- *Insult you or make you feel bad about yourself*
  *If yes, how many times did it happen within the one past month? (Once, more than once, it has never happened within past one month)*

The tool was translated and back-translated to and from Kiswahili, the language of communication in Tanzania. We pre-tested the tool in a similar setting as those planned for the survey to make sure questions are understandable and carry the intended meaning.

### Data analysis

Main data analyses involved descriptive statistics to characterize IPV (physical, sexual and emotional violence, each being an outcome variable). Looking for the association of each of the three components of IPV with selected variables, the test involved Chi-square. We used the logistic regression model to assess independent predictors of women reporting physical violence towards men perpetrated by their partners with three women’s demographic factors (age, marital status and education level) as independent variables. Since IPV behaviors may not be quite independent from one household to the other (clustered culturally or socially at village or street level), we used robust estimation of variances to account correlation between IPV rates in similar settings. The level of significance used was alpha = 0.05.

## Results

### Description of study participants

Study participants with complete data were 471 females. The majority, 317 (67.3 %) were married or cohabiting, only 115 (24.4 %) had at least secondary education. Their mean age was 32.2 (SD = 7.7) years. Other background characteristics are presented in Table 1.

**Table 1:** Distribution of study participants by background characteristics (*n* = 471)

Characteristic	Number (%)
Current age (years)	
25–29	161 (34.2)
30–34	100 (21.2)
35–39	82 (17.4)
40–44	61 (13.0)
45–50	67 (14.2)
Education	
Below secondary	356 (75.6)
Secondary and above	115 (24.4)
Current marital status	
Casual Partner	74 (15.7)
Married/Cohabiting	317 (67.3)
Divorced/Widow	80 (17.0)
Occupation^a^	
Small scale farming	140 (29.7)
Employed/Business	306 (30.1)
Other^b^	19 (4.1)

^a^Numbers do not add up to 471 because of missing responses

^b^Housewife, unemployed or student

**Fig. 1:**
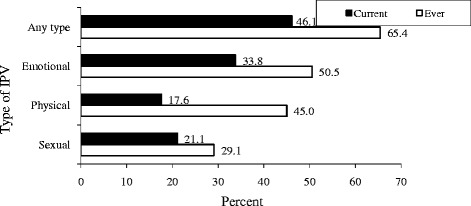
Proportion of women reporting current and lifetime types of intimate partner violence

Out of all women interviewed, 308 (65.4 %) [95 % CI = 60.9, 69.7], reported having experienced some form of IPV. Among those reporting to have experienced any form of IPV, 137 (46.1 %) reported recent (within one month) violence. The highest reported lifetime form of IPV was emotional violence, 238 (50.5 %) [95 % CI = 45.9, 55.1]. While 212 (45. 0 %) [95 % CI = 40.5, 49.6] reported lifetime physical violence, 137 (29.1 %) [95 % CI = 25.0, 33.4] of women reported lifetime sexual violence. Similarly, the reported current prevalence of physically, sexual and emotional violence was 17.6 % [95 % CI = 14.3, 21.4], 21.1 % [95 % CI = 17.6, 25.2] and 33.8 % [95 % CI = 29.5, 38.3] respectively (Fig. 1).

### Intimate Partner Violence (IPV)

In Table 2, we present results on the association between reported different forms of IPV by selected background characteristics (age, marital status and education level) of the respondents. All selected background characteristics were not associated with a woman reporting any form (physical, sexual or emotional) of IPV.

**Table 2:** Association between reporting intimate partner violence and characteristics of respondents

Type of IPV and characteristic of woman	Total Women (*n* = 417)	Number (%) reporting IPV, 137 (32.9 %)	χ^2^, *p*-value
Emotional violence			
*Age (Years)*			0.60, 0.96
25–29	161	82 (50.9)	
30–34	100	48 (48.0)	
35–39	82	42 (51.2)	
40–44	61	30 (49.2)	
45–50	67	36 (53.7)	
*Marital status*			
Casual partner	74	35 (47.3)	4.47, 0.11
Married/Cohabiting	317	154 (48.6)	
Previously married	80	49 (61.3)	
*Education status*			2.13, 0.35
None/Informal	16	6 (37.5)	
Primary	340	178 (52.4)	
Secondary and above	115	54 (47.0)	
Physical violence			
*Age (Years)*			3.22, 0.52
25–29	161	80 (49.7)	
30–34	100	40 (40.0)	
35–39	82	35 (42.7)	
40–44	61	25 (41.0)	
45–50	67	32 (47.8)	
*Marital status*			6.21, 0.05
Casual partner	74	30 (40.5)	
Married/Cohabiting	317	136 (42.9)	
Previously married	80	46 (57.5)	
*Education status*			3.66, 0.16
None/Informal	16	7 (47.8)	
Primary	340	162 (47.6)	
Secondary and above	115	43 (37.4)	
Sexual violence			
*Age (Years)*			1.84, 0.76
25–29	161	50 (31.1)	
30–34	100	29 (29.0)	
35–39	82	25 (30.5)	
40–44	61	18 (29.5)	
45–50	67	15 (22.4)	
*Marital status*			0.63, 0.73
Casual partner	74	20 (27.0)	
Married/Cohabiting	317	91 (28.7)	
Previously married	80	26 (32.5)	
*Education status*			2.67, 0.23
None/Informal	16	6 (37.5)	
Primary	340	104 (30.6)	
Secondary and above	115	27 (23.5)	

There is some evidence to suggest that women may also perpetrate violence against their partners, though rates are low. Out of 459 women who responded to this question, 32 (7.0 %) were affirmative that they ever physically abused their partners. Of these, five (15.6 %) reported recent (within past six months) physical abuse.

In Table 3 we present the prevalence of women’s perpetration to physical violence by their exposure to types of IPV. The prevalence of perpetration ranges between 10 and 11 % whether women are exposed to emotional, physical or sexual IPV. Among 237 women exposed to emotional violence, 24 (10.1 %) perpetrated physical violence against their male partners. Similarly, among 206 women who experienced intimate physical violence, 21(10.2 %) reported perpetration of physical violence against male partners too.

**Table 3:** Prevalence of women’s perpetration to physical violence by their exposure to types of IPV

Women exposed to IPV	Prevalence of perpetration Number (%)
Emotional violence	
Yes (*n* = 237)	24 (10.1)
No (*n* = 222)	8 (3.6)
Physical violence	
Yes (*n* = 206)	21 (10.2)
No (*n* = 253)	11 (4.3)
Sexual violence	
Yes (*n* = 136)	15 (11.0)
No (*n* = 323)	17 (5.3)

In Table 4, none of selected socio-demographic characteristics (maternal age, education and marital status) were found to independent predictors of women perpetration to IPV.

**Table 4:** Logistic regression on predictors of the female perpetration to physical violence

Predictor	Adjusted Odds Ratio^a^ (95 % CI)
*Age (Years)*	
25–29	Reference
30–34	1.5 (0.5, 4.5)
35–39	0.1 (0.0, 1.6)
40–44	1.5 (0.4, 5.2)
45–50	1.1 (0.3, 4.1)
*Marital status*	
Casual partner	Reference
Married/Cohabiting	0.5 (0.2, 1.5)
Previously married	1.0 (0.3, 4.1)
*Education status*	
Below secondary	Reference
Secondary and above	1.2 (0.5, 2.9)

^a^Including robust estimation of variances accounting for cluster sampling at village level

## Discussion

In this paper, we assessed intimate partner violence (IPV) directed to women and men as reported by women in urban areas of mainland Tanzania. The consequences of IPV are diverse; including social, physical, mental outcomes and death [21, 22]. Furthermore, IPV is also likely to hinder the economic and other developmental efforts in families and countries that experience these forms of violence not only in low-income but also in industrialized countries.

We found a relatively high (65 % for lifetime and 45 % for current) of reported any form of IPV towards women. Almost the same proportion has been reported among women in Southwest Ethiopia in 2012 [23]; higher than the proportion reported in Uganda but lower than that reported in Mozambique [24, 25]. Similarly, the proportions of women reporting physical and sexual violence were as high as 45 and 25 % respectively.

In this study, 45 and 29 % of women report exposure to intimate partner physical and sexual violence. These rates are slightly higher than those from one of the previous studies in Tanzania (DHS) that reports 36 and 21 % respectively [10]. Differences may be largely due to differences between the sample sizes between the two studies. The WHO multi-country study of 2005 reports the prevalence of life-time physical violence ranging from 33 to 47 % and sexual violence ranging from 23 to 31 % by an intimate partner among ever-partnered women in Tanzania [9]. In that study, one urban region (Dar es Salaam) and one rural region (Mbeya) were included in the study.

Although literature on physical violence perpetrated by women towards men is scanty [26], in urban Tanzania, the reported prevalence among ever-married women age 15–49 who report that they have initiated physical violence against their current or most recent husband was 2 % (overall and in rural areas) [10]. This rate is less than one-third of the current study (7 %). Another lower rate (4 %) was reported in Rwanda [27]. However, higher rates (21 %) of female perpetration to physical violence against a partner have been reported in South Africa (21 %), Ghana (16 %) and Uganda (41 %) [28, 29]. In any case, there is a possibility of underreporting of the incidents of violence directed towards men because culturally women may not admit that they themselves initiated violence. These findings suggest variability of IPV rates within and between settings in the sub-Saharan region. Variations in reporting IPV among women may be common due to the culture of silence when respondent feel shame, embarrassment and fear depending on their cultural settings and prevailing mood and social settings during interviews [30]. But lower rate of female perpetration than male suggest that women are at more elevated risks of IPV consequences than their counterparts. For example, studies have implicated IPV with increased risk for HIV infection because of high sexual behaviors such as unprotected sexual intercourse and infidelity [31–33].

In this study we did not find any association between the prevalence of IPV and demographic characteristics. Lack of associations suggests that IPV incidents are common in many settings and among many women and men regardless of their age, education status and their marital status. Other studies have found association between IPV and age, education and marital status. For example, several studies including Andersson et al., WHO and Iliyasu et al., found links between IPV with age, education level and marital status [9, 13, 14, 34]. With regard to the association between IPV and age and educational status, Abramsky et al. found young age, secondary education and formal marriage to be strongly associated with risk of experiencing IPV suggesting the need for targeted interventions to couples most at risk [15]. However, the previous reported associations between IPV and marital status or education could be a temporal such that more stringent analyses may be recommended.

In this study, we have a couple of potential limitations. First, we used face-to-face interviews rather than a self-administered tool. By using this technique, it is possible to introduce information bias because respondents might have been offering socially desirable answers. Second, although we tried to examine independent factors associated with IPV, the selected factors were not exhaustive and missed several social factors like alcohol consumption that has been documented elsewhere [35]. Third, asking about past events is influenced by memory capacity. Therefore, we cannot rule out the recall bias. Nevertheless, using the standard questions (for example questions that are used in big and validated surveys like DHS), including different urban communities, having well trained interviewers and privacy during the interviews all these strategies were likely to minimize under-reporting. Fourth, we were unable to establish the association between IPV and demographic characteristics; not because that there is no association, but maybe due the small sample size leading to Type I error. For that matter, caution must be taken when interpreting these results especially when a category has small numbers. Fifth, there are different ways to define and measure IPV. Such differences are likely to limit comparability of results from other studies. Furthermore, the study was cross-sectional and it was not possible to infer on causality. For example, does IPV against women cause women to retaliate against their partners or the other way round?

## Conclusions

To conclude, IPV is still a public health problem especially putting more women at risk. It should be more comprehended more hurting to find perpetrators of violence being one partner. In this study a substantial proportion of women report experiencing current and lifetime forms of IPV. More resources and programs should be mobilized by policymakers, public health experts and researchers are needed to address the problem of IPV. Furthermore, more data are required in this area so as to set up evidence-based strategies required for prevention and response to IPV.

## Abbreviations

CHAMPION, *Channeling Men’s Positive Involvement in a National HIV/AIDS Response;* CI, confidence intervals; DHS, Demographic and Health Survey; IPV, intimate partner violence; MAP, Men as Partners; OR, Odds Ratio; USAID, United States Agency for International Development
